# Kinematic and Kinetic Gait Principal Component Domains in Older Adults With and Without Functional Disability: A Cross-Sectional Study

**DOI:** 10.3390/jfmk10020140

**Published:** 2025-04-23

**Authors:** Juliana Moreira, Bruno Cunha, José Félix, Rubim Santos, Andreia S. P. Sousa

**Affiliations:** 1 CIR, E2S, Polytechnic of Porto, Rua Dr. António Bernardino de Almeida, 4200-072 Porto, Portugal; jmo@ess.ipp.pt (J.M.); rss@ess.ipp.pt (R.S.); 2 Research Center in Physical Activity, Health and Leisure, Faculty of Sports, University of Porto, 4200-450 Porto, Portugal; 3 CINTESIS@RISE, CINTESIS.UPT, Portucalense University, Rua Dr. António Bernardino de Almeida 541, 4200-072 Porto, Portugal; cun@isep.ipp.pt; 4 Department of Physiotherapy, Institute of Health of the North, Escola Superior de Saúde do Vale do Ave (ESSVA), Cooperativa de Ensino Superior Politécnico e Universitário (CESPU), Rua José António Vidal 81, 4760-409 Vila Nova de Famalicão, Portugal; jose.felix@ipsn.cespu.pt; 5 Department of Medical Sciences, University of Aveiro, Agras do Crasto, Campus Universitário de Santiago, 3810-193 Aveiro, Portugal

**Keywords:** gait, geriatrics, biomechanics, factor analysis, functional disability

## Abstract

**Objectives**: Gait kinematic and kinetic changes have been identified in older adults, highlighting the need to explore the principal age-related components and how these are associated with functional disability. This study aims to perform a factor analysis, including gait kinematic and kinetic parameters in older adults to establish determinant gait domains. Additionally, this study aims to identify which domains differentiate those without and with functional disability. **Methods**: Through a cross-sectional design, older adults aged 60 and over (n = 35 without and n = 25 with functional disability) were analyzed during overground gait. A principal component analysis (PCA) was used to determine principal components from gait parameters previously demonstrated to express age-related effects (spatiotemporal parameters, sagittal ankle moment and power, ground reaction forces peak, and tridimensional lower limb joints range of motion and positions at heel strike and toe-off). **Results**: *Pace*, *variability*, *propulsion*, *hip and knee control*, *transverse ankle control*, *asymmetry*, *sagittal ankle control*, *frontal ankle* control, *frontal hip control*, and *pre-swing control* domains explained 83.90% of the total gait variance in older adults. *pace* and *frontal hip control* distinguished individuals with disabilities. **Conclusions**: PCA identified ten gait domains in older adults. *Pace* and *frontal hip control* distinguished disabilities, revealing cautious walking patterns and weaker hip abductor strength.

## 1. Introduction

Declines in physical and cognitive functions in older adults stem from cumulative anatomical and physiological changes across multiple systems, which collectively impact functional mobility and overall performance [1,2]. This impact is determinant in daily activities when gait function declines, affecting functional independency [3]. Therefore, understanding age-related biomechanical changes during gait is essential for developing interventions to reduce disability risk.

Gait modifications in older adults have been studied through comparisons with younger adults. These comparisons highlighted changes across various parameters, including sagittal hip and ankle angle variability [4], center of pressure displacement [5], sagittal lower extremity kinematics, joint moments and powers at the ankle, and ground reaction forces (GRF) [6,7]. These findings suggest that age-related adaptations in gait cannot be adequately represented by a single parameter. Different decomposing methods for gait analysis have been used with several purposes [8,9]. Factor analysis methods can uncover the underlying structure within a set of biomechanical measures, reducing redundancies and highlighting the unique properties that define different performance aspects [10]. Dimensionality reduction methods, such as principal component analysis (PCA), have been proposed to classify gait characteristics into distinct domains based on discrete data or waveform analysis [11]. This approach offers a deeper understanding of the fundamental aspects of gait in older adults [10,12,13,14]. Different key domains have been proposed depending on the number of individual gait variables considered. Pace, rhythm, and variability were identified from eight individual gait variables [14]; phases of the gait cycle and base of support were added to the previous one when 23 individual gait variables were considered [13]. Complementarily, asymmetry and postural control domain parameters were added when adding these parameters and using step rather than stride characteristics [12]. More recently, it has been demonstrated that despite variability, pace, stability, complexity, time, and frequency components characterize older adults gait, only the time and frequency domains have been demonstrated to be affected by age [10]. Beyond these evolving results, it should be noted that the studies focus on key gait components of older adults, relying solely on spatiotemporal parameters. Evidence supports moderate standardized effects that demonstrate reductions in hip and ankle joint movements, peak ankle moments, power generation, and GRF in older adults [6,7]. Therefore, it is essential to understand how the inclusion of additional gait mechanics parameters, such as kinetics and angular kinematics, influences factor distribution.

Several metrics related to spatiotemporal gait parameters demonstrating age-related effects have been linked to an increased risk of disability in this population [15,16,17]. Specifically, gait speed was demonstrated to be correlated with hand grip strength [15], can predict incident disability [16], and its accelerated decline is also associated with disability, irrespective of baseline fast gait speed [17]. However, because this association was based on single spatiotemporal parameter comparisons, the comparative factor analysis of gait domains, including angular kinematics and kinetics, between older adults aging healthily and those with disabilities is also needed.

Building on these insights, this study aims to conduct a factor analysis, including gait tridimensional angular kinematic and kinetic and spatiotemporal parameters in older adults. The goal is to identify key domains of older adult gait and identify those that can differentiate individuals with and without disability.

To develop the comprehensive model of gait, the present study considered a sample of community-dwelling older adults to build a principal component model (PCM) that characterizes gait performance, including different gait parameters: (1) those spatiotemporal parameters included in previous PCA models (step time and length, variability and asymmetry, stride length, variability and asymmetry, swing and stance time, variability and asymmetry and step velocity, variability and asymmetry [12]); (2) joint kinematics (tridimensional lower limb joint range of motion (ROM) and positions at heel strike (HS) and toe-off (TO) in the frontal and transverse planes) and (3) kinematic and kinetic measures systematically reviewed (sagittal knee and ankle ROM, sagittal ankle angle at TO, peak hip extension and peak ankle plantar flexion, second peak of vertical ground reaction force (GRF second peak), anteroposterior GRF propulsion peak, sagittal peak ankle power generation and peak ankle dorsiflexion moment [6]). This integrative approach aims to establish a robust model of gait performance in older adults. Additionally, the scores of the principal components (PC) were compared between functional disability groups.

## 2. Materials and Methods

### 2.1. Study Design and Context

This cross-sectional study is part of a broader research initiative registered in the ClinicalTrials.gov database (identifier: NCT05611723) and adheres to the guidelines set forth by the Strengthening the Reporting of Observational Studies in Epidemiology [18]. The research was undertaken at the Center for Rehabilitation Research between 1 June 2022 and 31 March 2023. The study was approved by the Institutional Ethics Committee on 25 May 2022 (Registration Number: CE0064C). Data collection was executed from 1 June 2022 to 30 April 2023. All participants were provided with information regarding the study’s purpose and methodology, and they completed a form for informed consent.

### 2.2. Sample Selection and Characterization

A questionnaire was administered to gather demographic information, health conditions, prescribed medications, history of falls in the past 12 months, results from the Mini-Mental State Examination (MMSE), and the seven item short version of International Physical Activity Questionnaire (IPAQ). This questionnaire also included indicators to assess disability [19], particularly self-reported health (SRH), the Barthel Index of Activities of Daily Living (ADL), and the Lawton and Brody Instrumental Activities of Daily Living (IADL) Scale. Disability was further assessed through handgrip strength and the One-Leg Standing Test (OLST). Older adults were characterized as having a disability if they met two or more disability indicators.

Anthropometric measurements were taken, with body mass (kg) assessed by bioimpedance analysis (Tanita Inner Scan BC-601, Tanita Europe B.V., Hoogoorddreef 56E; 1101 BE; Amsterdam; The Netherlands) and height measured using a seca^®^ 222 stadiometer (seca—Medical Scales and Measuring Systems^®^, Birmingham, UK), with 1 mm precision. Information on prescribed medications was collected, with polypharmacy defined as the use of five or more prescription medications [20]. The MMSE [21] was administered to evaluate cognitive function, which includes assessing temporal and spatial orientation, short-term memory, calculation, coordination, language, and visuospatial skills. Total MMSE scores range from 0 to 30, with scoring criteria adjusted for literacy levels: 22 for 0–2 years of literacy, 24 for 3–6 years, and 27 for 7 or more years [22]. Self-reported physical activity was assessed by the seven item short version of IPAQ, considering moderate and vigorous physical activity [23].

Overall SRH was assessed through the question, “In general, how do you rate your health today”? with response options of “Very bad”, “Bad”, “Fair”, “Good”, and “Very Good”. SRH was then dichotomized into “Good” (responses of “Good” or “Very Good”) and “Poor” (responses of “Fair,” “Bad,” or “Very Bad”) categories [24]. The Barthel Index was used to evaluate participants’ independence in ADL [25,26]. The scoring system of 0 indicating complete dependency and 20 indicating functional independence was used as this scoring is correct for an uneven impression of accuracy [27]. Independence in IADL was assessed using the Lawton and Brody Scale [28], with total scores ranging from 7 to 23 [29]. Handgrip strength was measured with a Jamar^®^ Plus+ Digital dynamometer (Performance Health Supply, Cedarburg, WI, USA), with participants seated, shoulder adducted, elbow flexed at 90 degrees and unsupported, forearm in a neutral position, and wrist extended to 30 degrees, following guidelines by the American Society of Hand Therapists [30]. The OLST was performed on the preferred leg, with eyes open, to assess balance in seconds [31]. Timing ceased when the participant lost balance (for example, when the flexed leg touched the ground), with a maximum duration of 60 s [31].

### 2.3. Procedures

Following the assessment of characterization data and functional disability features, the next procedure was the participants preparation for the data collection with the optoelectronic system. The kinematic and kinetic data were collected synchronously using the Qualisys Track Manager^®^ (Qualisys AB^®^, Göteborg, Sweden) with eleven optoelectronic cameras (eight Oquos500, three MiqusM3), one Miqus video camera (Qualisys AB^®^, Sweden), and two force plates (FP4060-08/10, Bertec^®^; Columbus, OH, USA) at a sampling frequency of 100 Hz [32]. A sampling frequency of 100 Hz was set [32], respecting the Nyquist theorem on sampling frequency for the gait kinetic variables, ensuring that the signal was accurately portrayed in the time domain without missing peak values [33,34]. A marker setup adapted from the collaboration between Istituto Ortopedico Rizzoli and other authors [35,36,37], augmented with the head segment [38] and Rab Upper Extremity Model [39], was used for data acquisition (Table 1).

**Figure 1:**
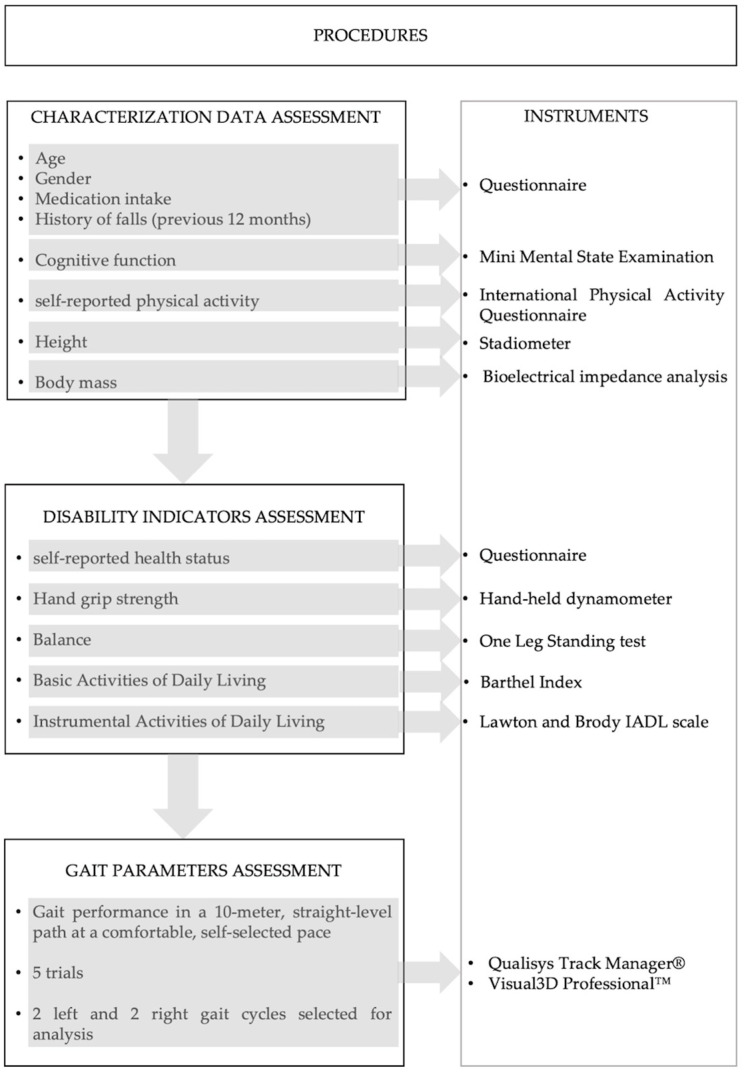
Flowchart of study procedures.

To assess overground gait, participants were instructed to walk a 10 m, straight-level path at a comfortable, self-selected pace. Sufficient practice trials were provided to ensure a natural gait pattern. Participants achieved correct foot placement with full-foot contact on a force plate during five successful overground trials [40]. The force plates were aligned in sequence to capture the central gait cycles, thus excluding gait initiation and termination phases [41]. The procedures are summarized in Figure 1.

### 2.4. Data Processing

Data were processed using both Qualisys Track Manager (Qualisys AB^®^, Sweden) and Visual3D Professional™ (Has-Motion, Inc., Kingston, ON, Canada). To eliminate minor random digitization errors and certain soft-tissue artifacts, a Butterworth low-pass filter with a cut-off frequency of 6.0 Hz was applied [32]. A gait cycle was defined as the interval between two consecutive HSs of the same foot. The HS was identified when the vertical component of the GRF reached 20 Newtons (N), while TO was defined as the point at which this force fell to 20 N or below [42].

### 2.5. Gait Parameters for PCA

To conduct the factor analysis on spatiotemporal parameters, the rationale proposed by Lord and colleagues [12] was adhered to. The inclusion of a sufficient number of gait characteristics was emphasized to ensure the model accurately represents the underlying construct of gait while minimizing parameter redundancy. Specifically, the study relied on step characteristics due to their heightened reliability, maintained original measurement units, and incorporated measures of step asymmetry. Furthermore, the analysis utilized standard deviation (SD) instead of the coefficient of variation, defined as mean/SD × 100, as it provides greater clarity for interpretation [12]. The spatiotemporal measures were included in a preliminary model that replicated the work proposed by Lord and colleagues [12]. This model included in the analysis step swing, and stance time, all measured in seconds; step and stride length, measured in meters; and step velocity, computed in meters per second (Table 2). Variability and asymmetry were computed for these measures, with the exception of asymmetry in step velocity [12] (Table 2).

A second primary PCA model focused on lower limb joint ROM and positions at HS and TO in frontal and transverse planes.

The comprehensive PCA model of gait included, in addition to the parameters with loadings above 0.7 on the previous two models, the angular kinematic and kinetic measures identified in the systematic review and meta-analysis of gait mechanics in young and older adults [6]. The measures included had to demonstrate a statistically significant standardized moderate to large effect of age on gait. Specifically, kinematic measures included sagittal knee and ankle ROM, sagittal ankle angle at TO, peak hip extension, and peak ankle plantar flexion. Kinetic measures included the second peak of vertical GRF (GRF second peak), anteroposterior GRF propulsion peak, sagittal peak ankle power generation, and peak ankle dorsiflexion moment (Table 2).

Measurements of lower limb joint angles (hip, knee, and ankle) were recorded in degrees and calculated using the Cardan sequence, which assumes that the x-axis is in the medial-lateral direction (positive flexion and negative extension movements), the y-axis is anterior-posterior (positive abduction and negative adduction movements), and the z-axis is in the up-and-down or axial direction (positive longitudinal internal and negative external rotation) as recommended by the International Society of Biomechanics for the lower limb [32,43].

The method of inverse dynamics was employed to integrate kinematic data and external GRF in order to derive joint moments and powers at the ankle joints [44]. Joint moment was normalized to the subject’s mass. The joint power was computed by combining the joint moments (Nm/kg) and joint angular velocities (rad/s), with positive values for joint power showing power generation (related to concentric muscle activity) and negative values showing power absorption (related to eccentric muscle activity) [32], with all measures normalized to the subject mass. Definitions for all gait parameters included in factor analysis are presented in Table 2.

**Table 2:** Definitions of gait parameters included in the factor analysis.

Type	Gait Outcome	Definition
Spatiotemporal parameters	Step length (meters)	Average distance, in meters, between the position of the right/left foot in the heel strike and the position of the left/right foot in the next heel strike [45].
	Step velocity (meters/second)	The calculation involves determining the length of each step divided by the corresponding step time for both the right and left sides [45].
	Step time (seconds)	Average time, in seconds, between the right/left heel strike and the left/right heel strike [45].
	Swing time (seconds)	Average time, in seconds, between the right/left toe-off and the right/left heel strike [45].
	Stance time (seconds)	Average time, in seconds, between the right/left heel strike and the right/left toe-off [45].
	Stride width (meters)	Distance between the proximal end position of the foot at ipsilateral heel strike to the proximal end position of the foot at the next ipsilateral heel strike [45].
Joint kinematics measures	Sagittal/frontal/transverse hip ROM (degrees)	Range of motion of hip, knee, and ankle angle, in sagittal/frontal/transverse plane, measured in degrees, obtained by subtracting the minimum from the maximum value along the gait cycle.
	Sagittal/frontal/transverse knee ROM (degrees)	
	Sagittal/frontal/transverse ankle ROM (degrees)	
	Peak hip extension (degrees)	Maximum value, in degrees, of hip extension, flexion, and ankle plantar flexion along the gait cycle.
	Peak hip flexion (degrees)	
	Peak ankle plantar flexion (degrees)	
	Sagittal/frontal/transverse hip angle at HS (degrees)	Value, in degrees, of hip, knee, and ankle sagittal/frontal/transverse angles at the events of heel strike or toe-off.
	Sagittal/frontal/transverse hip angle at TO (degrees)	
	Sagittal/frontal/transverse knee angle at HS (degrees)	
	Sagittal/frontal/transverse knee angle at TO (degrees)	
	Sagittal/frontal/transverse ankle angle at HS (degrees)	
	Sagittal/frontal/transverse ankle angle at TO (degrees)	
Variability measures	Step time variability (s)	The combined standard deviation of left and right steps was calculated by taking the square root of the mean variance of the left and right steps according to the formula: $\mathrm{SDLeft}\&\mathrm{Right}=\frac{\sqrt{Variance\;Left\;steps\;+\;Variance\;Right\;Steps}}{2}$ [46]
	Stance time variability (s)	
	Swing time variability (s)	
	Step velocity variability (m/s)	
	Step length variability (m)	
Asymmetry measures	Step time asymmetry (% gait cycle)	The comparison of the right side parameter to the left side parameter using the formula: $100\;*\;|\ln\frac{Right\;Side\;parameter}{Left\;Side\;parameter}|$ [47]
	Swing time asymmetry (% gait cycle)	
	Stance time asymmetry (% gait cycle	
	Step length asymmetry (% gait cycle)	
Kinetic measures	Vertical GRF second peak (BW)	Second maximum value of vertical ground reaction force, measured in Newtons, normalized to participant mass measured in kilograms (Kg) multiplied by acceleration due to gravity (9.81 m/s^2^) [6,45].
	Anteroposterior GRF peak (BW)	Maximum value of antero-posterior ground reaction force, measured in Newtons, normalized to participant mass measured in kilograms (Kg) multiplied by acceleration due to gravity (9.81 m/s^2^) [48].
	Ankle peak dorsiflexion moment (Nm/Kg)	Moment of force equivalent to the sum of all moments of force acting across the ankle joint, normalized to participant mass measured in kilograms (Kg) [33,45].
	Sagittal peak ankle power generation (W/Kg)	The joint power was computed by multiplying the joint moments (Nm/kg) and joint angular velocities (rad/s), normalized to participant mass measured in kilograms (Kg) [45].

### 2.6. Data Analysis

The demographic and clinical characteristics of the participants were analyzed using means and standard deviations, along with frequency counts to describe variables such as sex, history of falls in the past 12 months, polypharmacy, and SRH status. Between-group comparisons of demographic and clinical characteristics were conducted using either Mann–Whitney tests or independent sample *t*-tests, depending on the normality of the distribution. Chi-squared tests were employed to assess between-group associations concerning categorical variables, specifically sex, history of falls, and SRH status.

PCA was conducted on kinematic and kinetic parameters, primarily in Lord and colleagues model replication and in the model, including lower limb frontal and transverse ROM and positions at HS and TO. A correlation matrix was used to ensure that each variable contributed equally to the analysis. The correlation matrix was constructed based on standardized values, with each variable divided by its SD to normalize the data, resulting in a mean of 0 and an SD of 1 for all variables [49].

Potential outliers in the data were manually examined to ensure they would not exert an undue influence on the PCA results. No exclusion of outliers was required in any of the PCA models performed. The suitability of the dataset for PCA was assessed using the Kaiser–Meyer–Olkin (KMO) as a measure of sampling adequacy and Bartlett’s Test of Sphericity, which evaluates the appropriateness of the data for reduction. A significant Bartlett’s Test result (*p* < 0.05) and KMO values exceeding 0.5 were used to determine the fitness of the data for PCA. PCs accounting for the majority of the variance were extracted based on eigenvalues greater than 1. To enhance the interpretability of the components, a Varimax rotation was applied. The resulting rotated component matrix was analyzed to identify clusters of variables that loaded onto the same components, with an emphasis on variables exhibiting high loadings (loading > 0.7, in accordance with Hair et al. [50]) for each component. The same procedures were applied to the comprehensive model [49]. This included the measures with loadings > 0.7 of the Lord and colleagues [12] model replication, complementary PCA model on lower limb positions in frontal and transverse planes, and the kinematic and kinetic measures with statistically significant standardized moderate and high effect of age on gait, by Boyer et al., 2017 [6]. The differences between older adults with and without disability indicators across each of the kPCs of the comprehensive gait PCM were assessed using the non-parametric Mann–Whitney U test, with a significance level set at 0.05. All analyses were performed using IBM SPSS Statistics for Macintosh, Version 29.0.2.0.

### 2.7. Ethical Considerations

The study adhered rigorously to the principles outlined in the Declaration of Helsinki. All participants received information about the study’s purpose and methodology, and they completed an informed consent form. The study was submitted to the ESS, Polytechnic of Porto Institutional Ethics Committee on 27 April 2022, and obtained authorization on 25 May 2022 (Approval No. CE0064C).

## 3. Results

### 3.1. Sample Characterization

**Figure 2:**
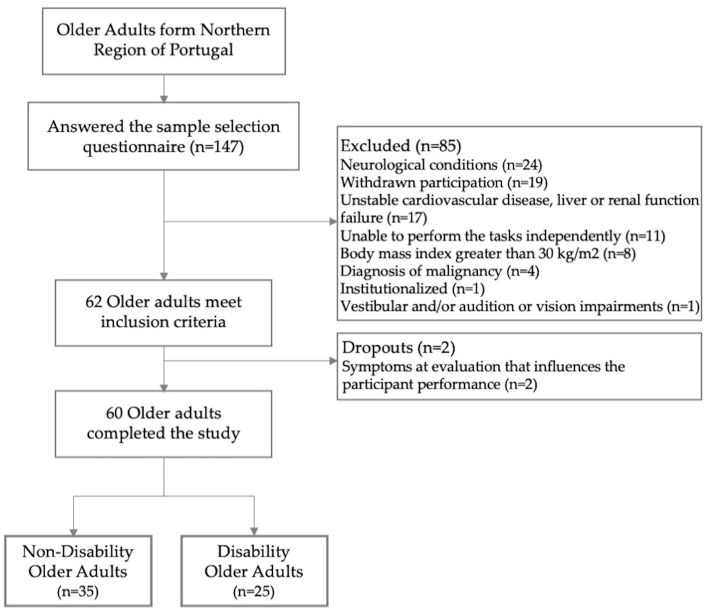
Flowchart of sample selection process.

Out of 147 older adults who completed the eligibility questionnaire, 62 individuals enrolled in the study (Figure 2). During the evaluation, two participants displayed symptoms that impaired their performance, resulting in a final sample of 60 participants included in the analysis.

The demographic and clinical data showed no differences between older adults without and with disability, except for polypharmacy, in which almost half of the group of older adults with disability took five or more medicines per day (Table 3). The groups presented significant differences in all disability indicators.

### 3.2. PCA Primary Models

The replication of Lord and Colleagues factor analysis [12] confirmed the adequacy of the current study’s sample for dimensionality reduction (KMO = 0.647 and Bartlett’s Test of Sphericity *p* < 0.001). The PCA model identified four components (explaining 79.3% of total variance), with the pace and rhythm gait spatiotemporal parameters loading onto the same component (see Supplementary Table S1). The variability component defined by Lord et al. included parameters such as step velocity variability, step length variability, and step width variability. Similarly, the present model included step velocity variability in the variability component but differed in including step time variability as well as swing and stance time variability. Step length, which Lord and colleagues associated with the *pace* component, loaded onto the postural control component in this study. Specifically, the *postural control* component in the present model encompassed all spatiotemporal parameters related to length and width, including step length asymmetry, step length, step length variability, and step width. Accordingly, the parameters with loadings above 0.7 and thus included in the comprehensive model of gait were step, stance, swing time, step velocity, step time, stance time, step velocity and swing time variability, swing time, step time and stance time asymmetry.

The complementary factor analysis, including lower limb frontal and transverse ROM and positions at HS and TO, demonstrated suitable dataset characteristics for PCA (KMO = 0.535 and Bartlett’s Test of Sphericity *p* < 0.001). Six components were retained, collectively explaining 79.19% of the data variance (see Supplementary Table S2).

The first component primarily reflected ankle joint control in the transverse plane, capturing joint positions at TO and HS, as well as the transverse knee angle at TO, all with loadings exceeding 0.8. The second component included the transverse hip angle at HS and TO, along with the frontal knee angle at TO.

The third PC was defined by the frontal hip ROM, with a loading above 0.7, while the fourth PC was associated exclusively with the frontal ankle angle at HS and TO, both having loadings above 0.8. The fifth component comprised the frontal hip angle at TO and HS, whereas the sixth component was defined solely by the transverse hip ROM.

In summary, the parameters with loadings greater than 0.7 included the comprehensive model were the ankle joint control in the transverse plane (at TO and HS), as well as the transverse knee angle at TO, transverse hip angle at HS and TO, frontal knee angle at TO, frontal hip ROM, frontal ankle angle at HS and TO, frontal hip angle at TO and HS, and transverse hip ROM.

### 3.3. Comprehensive Model of Gait of Older Adults and Differences Between Those with and Without Functional Disability

**Figure 3:**
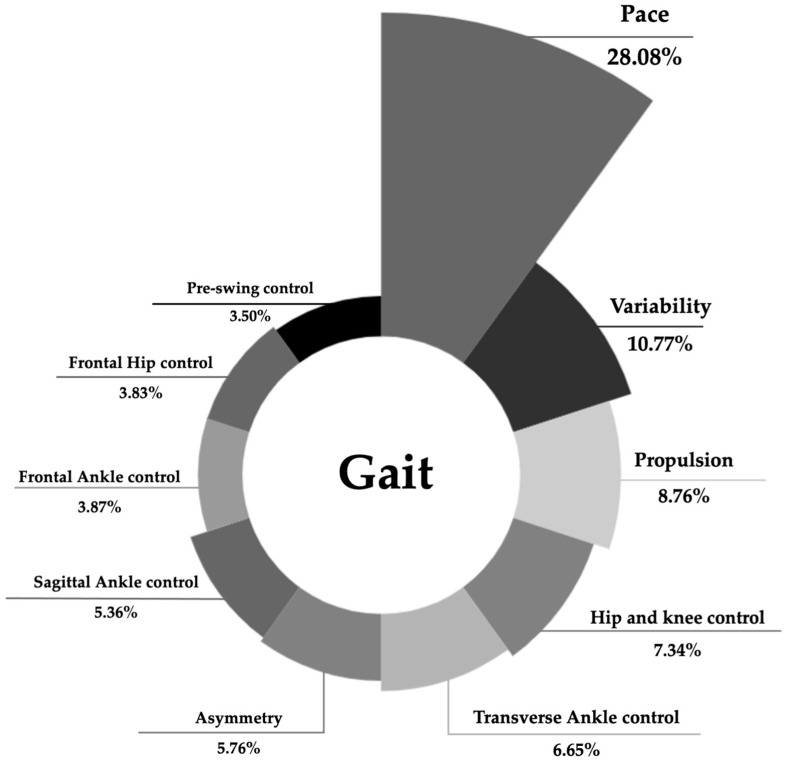
Total variance explained by the principal components of the comprehensive model of older adult gait.

The comprehensive model of older adults gait included 32 spatiotemporal, kinetic, and kinematic variables (see Table 4). This comprehensive model retained ten PCs, named according to the representative set of parameters, explaining approximately 83.90% of total variance. The suitability of the dataset for PCA was ensured by a KMO = 0.597, as well as sampling adequacy by the Bartlett’s Test of Sphericity *p* < 0.001. The pace, variability, and asymmetry components were included in this model, explaining 28.08%, 10.77%, and 5.75% of total variance, respectively (Figure 3). The propulsion component (8.76%) gathered the kinetic measures of the ankle, particularly the sagittal peak ankle power, the vertical GRF second peak, and the AP GRF peak. The sagittal ankle ROM is also included in the propulsion component but with loading < 0.7. The fourth PC included the transverse hip angle at TO and HS and the frontal knee angle at TO.

Additionally, the model grouped the variables of control of the ankle on transverse, sagittal and frontal plane, explaining 6.65%, 5.36% and 3.87% of total variance, respectively. Finally, the component that explained less variance was the pre-swing control (3.50%) that loaded higher than 0.7 only the ankle peak dorsiflexion moment.

**Figure 4:**
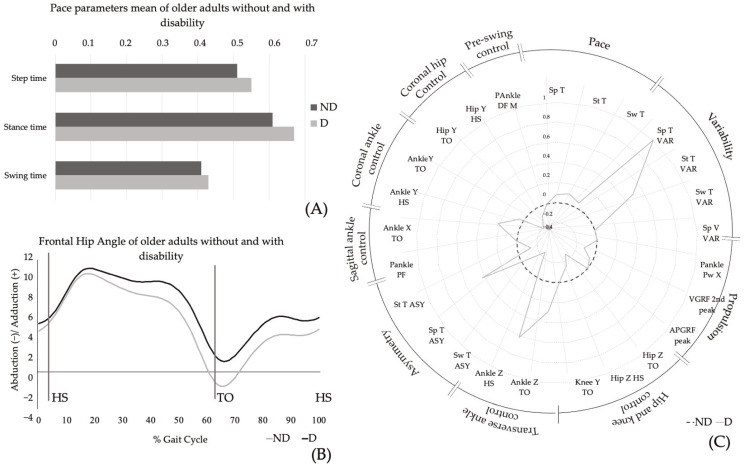
Plots of parameters included in principal components with significant differences in older adults without and with functional disability: (**A**) Pace parameters (step, stance, and swing time) means for older adults without (ND) and with (D) functional disability; (**B**) frontal hip angle measured in degrees along the gait cycle of older adults without (ND) and with (D) functional disability; (**C**) standard deviations of the functional disability group (D) relative to their non-disabled counterparts (ND) for each parameter with loading > 0.7 of the comprehensive model of older adults gait. Sp T—step time; St T—stance time; Sw T—swing time; Sp T VAR—step time variability; St T VAR—stance time variability; Sw T VAR—swing time variability; Sp T VAR—step velocity variability; Pankle PW X—sagittal peak ankle power; VGRFv2nd peak—vertical GRF second peak; APGRF peak—AP GRF peak; Hip Z TO/HS—transverse hip angle at TO; Knee Y TO—frontal knee angle at TO; Ankle Z TO/HS—transverse ankle angle at TO/HS; Sw T ASY—swing time asymmetry; Sp T ASY—step time asymmetry; St T ASY—stance time asymmetry; Pankle PF—ankle peak plantar flexion; Ankle X TO—sagittal ankle angle at TO; Ankle Y HS/TO—frontal ankle angle at HS/TO; Hip Y TO/HS—frontal hip angle at TO/HS; PAnkle DF M—ankle peak dorsiflexion moment.

Differences were identified between the PC scores of older adults without and with functional disability in the *pace* and *frontal hip control* components (see Table 2). The mean values of gait parameters included in *pace* component (swing, stance, and step time) are illustrated in Figure 4A, highlighting significant group differences in stance time. Figure 4B depicts the frontal hip angle throughout the gait cycle, showing that while the overall behavior of older adults with functional disability is similar to their counterparts without functional disability, the former exhibit a more global adduction hip position and reduced ROM. These differences, when represented by the standard deviations of the functional disability group relative to their non-disabled counterparts, underscore the significance of additional measures, such as step time variability, transverse ankle angle at HS, and stance time variability (Figure 4C).

## 4. Discussion

This study used PCA to identify specific domains of gait in older adults, encompassing angular kinematic, kinetic, and spatiotemporal parameters. Additionally, it aimed to distinguish these domains between older adults with and without functional disability.

The rationale for this study builds on previous research that applied factor analysis to identify gait domains in older adults [12,13,14]. However, previous studies were limited to spatiotemporal parameters, generally identifying three to five distinct gait domains [12,13,14]. More recently, Hagoort and colleagues advanced this approach by analyzing 27 gait measures, incorporating not only spatiotemporal parameters but also metrics that reflect the dynamic aspects of walking on a sample of healthy young and older adults [10]. In the present study, angular kinematic and kinetic measures of gait mechanics were included to provide a comprehensive understanding of older adults’ performance and to examine how functional disability development in later life impacts this performance. The initial step involved replicating the model proposed by Lord and colleagues, which identified four components. However, the component comprising stride width, step length, and its asymmetry and variability had loadings below 0.7, leading to its exclusion from the comprehensive model of this study. The lower loadings of length-related measures may be attributed to the greater representativeness of stride length, compared to step length, in capturing age-related changes. Indeed, factor analyses using stride length measures have shown higher loadings for these parameters [10,14]. Similarly, the low loading of width-related measures aligns with previous research indicating that these parameters remain relatively stable across aging [51].

Additionally, gait spatiotemporal parameters related to pace and rhythm loaded onto the same component. As emphasized by Lord and colleagues, gait characteristics do not always load onto identical domains [12]. This inconsistency was observed in the other three components. For instance, all spatiotemporal parameters related to length and width were grouped within the postural control domain. This may reflect the strong interrelationship among spatiotemporal parameters and suggests that these measures alone offer a reductive perspective on gait performance [12,13]. To address this limitation, angular kinematic and kinetic parameters with statistically significant standardized moderate to high age effects, as identified by Boyer et al., 2017, were included. However, these parameters predominantly focus on the sagittal plane [6]. Studies on gait analysis in older adults often prioritize sagittal plane parameters due to their clinical relevance, ease of measurement, and lower error rates compared to measurements in the frontal or transverse planes [33,52]. While the sagittal plane provides critical insights into walking dynamics and the diagnosis of mobility impairments, there is growing recognition that multi-plane analysis is essential for a more comprehensive understanding of age-related changes in gait [6,33]. Consequently, the second step involved analyzing the loading of joint positions in the frontal and transverse planes.

Therefore, the comprehensive model incorporated 32 variables, which were grouped into ten distinct domains. Among these, the domain that accounted for the largest proportion of variance was *pace*, encompassing step, stance, and swing time, as well as step velocity, but with loading lower than 0.7. These findings align with previous factor analyses [12,13,14], where *pace* and *rhythm* components consistently explained a substantial portion of the variance. This predominance may be attributed to the cautious gait behavior commonly observed in older adults, as identified in prior research [51], but also to the impact of angular kinematic and kinetic adaptations of these variables [53]. The second component identified was *variability*, which included all parameters used to characterize gait variability, such as step, stance, and swing time variability and step velocity variability. The consistency of this domain can be attributed to the initial step of excluding parameters with non-representative loadings. The significance of these measures is crucial, as gait variability metrics have the potential to detect changes in the motor control system’s capacity to respond to environmental and task constraints [54], even when the subsystems contributing to gait variability exhibit only subtle changes [55]. Moreover, the stance time variability presents as an independent predictor of future mobility disability [56].

The kinetic measures associated with the push-off phase of the gait, specifically, sagittal peak ankle power, the second peak of vertical ground GRF, and the anterior-posterior GRF peak, were grouped into a single component labeled *propulsion*. This novel component aligns with and reinforces previous findings in the literature regarding the decline in forward propulsion observed in older adults, particularly the reduction in ankle push-off power [57]. While this reduction is typically reported in individuals aged 70 years or older [57], the present study includes older adults aged 60 and above; this component accounts for 8.76% of the total variance, suggesting that these changes may begin earlier. The literature attributes these declines to the functional consequences of sarcopenia and plantar flexor muscle weakness, as well as the underutilization of available muscular capacity [58]. These age-related declines, combined with reduced flexibility, are reflected also in the *sagittal ankle control* component, which includes the ankle peak plantar flexion and the sagittal ankle angle at TO [7].

The importance of the control in the transverse plane is elucidated by the *hip and knee control* and *transverse ankle control* domains. These components encompass the transverse hip and ankle joint angles at HS and TO gait events, parameters that have been minimally explored in the existing literature [7]. However, the present results suggest that these aspects play a crucial role in the gait biomechanics of older adults, offering valuable insights into their functional mobility and potential gait adaptations.

In line with this, the joint behavior of older adults in the frontal plane was characterized by the *frontal ankle control* and *frontal hip control* domains. The pattern of elevation and depression of the iliac crests reflects the frontal plane hip motion, potentially indicating hip abductor weakness in older adults [33]. The gluteal muscles of the hip are particularly susceptible to adipose infiltration and reduced muscle fiber quality [59], which may explain the prominence of this component in describing the gait characteristics of older adults. Notably, the *frontal hip control* PC scores revealed significant differences between older adults with and without functional disability. Consistent with these findings, maximum voluntary isometric strength and rate of force generation of the hip abductors have been shown to possess good diagnostic accuracy in distinguishing between older fallers and non-fallers [60]. More importantly, hip abduction strength has been identified as a predictor of disability [61]. These physiological changes may be exacerbated in older adults with functional disability, who exhibit a more adducted hip position and reduced ROM throughout the gait cycle compared to their non-disabled counterparts. Moreover, hip adduction has been identified as one feature that might be used in a clinic to distinguish between deficits of age compensation [62]. Building on this, sagittal plane adaptations also reveal critical compensatory mechanisms that distinguish aging patterns. Notably, age-related increases in gluteus maximus activation suggest a distal-to-proximal shift in muscle function, where reduced ankle power (supported in this study by the third component) is compensated for by enhanced hip extensor recruitment [63]. This shift likely contributes to gait preservation despite declining distal joint performance. However, in contrast to the increased extensor activity, older adults exhibit decreased activation of key hip flexors such as the iliacus and psoas during late stance, indicating a change in neuromuscular control strategies. When considered alongside the observed frontal plane deficits—such as reduced hip abductor strength and increased adduction—these sagittal plane findings emphasize a multidimensional reorganization of hip mechanics with aging. Importantly, the combination of reduced frontal plane control and altered sagittal plane muscle activation may compound mobility limitations in older adults with functional impairments.

Similarly, the *pace* domain, which encompasses step, stance, and swing time, also demonstrated differences between older adults with and without disabilities. Older adults with functional disability exhibited increased durations of these parameters, aligning with evidence that identifies gait speed as a strong indicator of disability and other adverse outcomes [64,65]. Among these parameters, the step stance time showed the greatest disparity between groups, suggesting that older adults with disabilities adopt a strategy of prolonging the support phase of each limb during gait. This strategy is further highlighted by the variability of this parameter, as represented by the standard deviations of the functional disability group relative to their non-disabled counterparts. Indeed, literature underscores the importance of step stance time variability, identifying it as an independent predictor of mobility disability and a risk factor for future disability [56]. Additionally, differences in standard deviations emphasized the variability of step time and the transverse ankle angle at HS, further supporting the critical role of the step time variability parameter in assessing mobility and predicting disability in older adults [66] and the need to further explore the transverse ankle kinematics [7].

The results of this study should be interpreted in light of its limitations, particularly the sample size. Nonetheless, measures of sampling adequacy and the appropriateness of the data for dimensionality reduction were rigorously ensured for all models employed. In contrast, prior studies often do not report on sample adequacy or data suitability for reduction [12,13,14], an issue highlighted both in this scope and in related fields [67]. Furthermore, the sample size in this study is larger than that of many biomechanical studies, which frequently involve small cohorts [6]. The retaining loadings were appropriately aligned with the sample size [50], addressing a common methodological shortcoming in previous studies, where loadings > 0.5 were retained even with insufficient sample sizes [10]. Additionally, comparing the parameter values with normative data for this population would enhance the interpretability of the gait parameter domains and the differences observed between functional disability groups.

Future studies should aim to replicate this model using larger sample sizes, which could enhance the robustness of the model while enabling the integration of additional variables. For instance, although this study focused on kinetic measures identified through a systematic review, considering the integration of frontal and transverse plane kinetics could further deepen our understanding of gait in older adults. These measures, traditionally examined in contexts such as stepping [68] or stair negotiation [69], are increasingly recognized as critical parameters of gait [70]. This approach would allow for a more comprehensive understanding of the underlying mechanisms and contribute to the generalizability of the findings. Given the consistency of the *variability* component for the spatiotemporal parameters, including measures of joint kinematics and kinetic variability observed to vary with age [4], could provide valuable insights into the motor system’s ability to adapt to diverse environmental and task constraints and consequently the capacity for adaptation [54].

## 5. Conclusions

Older adults angular kinematic, kinetic, and spatiotemporal parameters gait performance of older adults can be characterized into ten domains, particularly *pace*, *variability*, *propulsion*, *hip and knee control*, *transverse ankle control*, *asymmetry*, *sagittal ankle control*, *frontal ankle control*, *frontal hip control*, and *pre-swing control*. The *pace* and *frontal hip control* domains differentiate individuals with and without functional disability, suggesting that older adults with functional disability exhibit more cautious gait behaviors and greater weakness in abductor musculature compared to their non-disabled counterparts.

Prospective research should consider replicating this model with larger sample sizes to strengthen its validity, refine its precision, and integrate additional variables to potentially offer deeper insights into the multifaceted nature of gait biomechanics and disability in older adults.

## Figures and Tables

**Table 1:** Anterior and posterior list of markers.

	Marker Acronym	Description
Anterior view	*L/RALH*	Left/right anterior head
	*L/RCAJ*	Left/right acromion
	*SJN*	Deepest point of incisura jugularis
	*SXS*	Xiphoid process, the most caudal point of the sternum
	*L/RA 1, 2, 3*	Left/right cluster arm *1, 2, 3*
	*L/RFA 1, 2, 3*	Left/right cluster forearm *1, 2, 3*
	*L/RRAD*	Left/right radio-styloid process
	*L/RULN*	Left/right ulna-styloid process
	*L/RIAS*	Left/right anterior superior iliac spine
	*L/RFTC*	Most lateral prominence of the greater trochanter
	*L/RTH 1, 2, 3, 4*	Left/right cluster thigh *1, 2, 3, 4*
	*L/RFLE*	Most lateral prominence of the lateral femoral epicondyle
	*L/RFME*	Most medial prominence of the medial femoral epicondyle
	*L/RFAX*	Proximal tip of the head of the fibula
	*L/RTTC*	Most anterior border of the tibial tuberosity
	*L/RSK 1, 2, 3, 4*	Left/right cluster shank *1, 2, 3, 4*
	*L/RFAL*	Lateral prominence of the lateral malleolus
	*L/RTAM*	Most medial prominence of the medial malleolus
	*L/RFM5*	Dorsal margin of the fifth metatarsal head
	*L/RFM2*	Dorsal aspect of the second metatarsal head
	*L/RFM1*	Dorsal margin of the first metatarsal head
	*L/RDR*	Left/right distal radius
	*L/RDU*	Left/right distal ulna
Posterior View	*L/RPH*	Left/right posterior head
	*CV7*	Spinous process of the seventh cervical vertebra
	*TV2*	Second thoracic vertebra
	*TV7*	Midpoint between the inferior angles of the two scapulae
	*LV1*	First lumbar vertebra
	*LV3*	Third lumbar vertebra
	*LV5*	Fifth lumbar vertebra
	*L/RIPS*	Left/right posterior superior iliac spine
	*L/RFCC*	Aspect of the achilles tendon insertion on the calcaneous
	*L/RLELB*	Left/right lateral epicondyle of humerous
	*L/RMELB*	Left/right medial epicondyle of humerous
	*L/RMH*	Left/right medial head of fifth metacarpal
	*L/RLH*	Left/right lateral head of fifth metacarpal

**Table 3:** Demographic and clinical characterization of the sample. Data represented by the mean and standard deviation for ordinal variables and frequencies for nominal variables. The *p*-value reflects the comparison between older adults without disability (ND) and older adults with disability (D) by ^(a)^ Mann–Whitney U test, ^(b)^ Chi-square test, and ^(c)^ Independent samples *t*-test.

Demographic and Clinical Data		ND (n = 35)	D (n = 25)	*p*-Value (Test Value)
Age (years)		66.34 ± 5.60	68.60 ± 6.77	0.147(534) ^(a)^
Gender (n female; %)		19; 54.29	19; 76	0.085(2.961) ^(b)^
BMI (kg/m^2^)		25.22 ± 3.08	26.02 ± 2.66	0.298(−1.049) ^(c)^
History of the fall, previous 12 months (n fallers/%)		11 (31.4)	11 (44)	0.469(0.525) ^(b)^
Polypharmacy (n polymedicated/%)		2 (5.71)	11 (44)	<0.001(12.595) ^(b)^
Cognitive function (MMSE score)		28.94 ± 1.31	28.68 ± 1.49	0.495(394) ^(a)^
Self-reported physical activity (IPAQ MET-min/week)		3186.46 ± 2964.91	3519.66 ± 2822.11	0.509 (393.5) ^(a)^
Disability indicators				
Self-reported health	poor	8 (22.86)	21 (84)	<0.001(21.832) ^(b)^
	good	27 (77.14)	4 (16)	
Hand grip strength (kg)		36.59 ± 39.86	25.07 ± 7.54	0.018(279.5) ^(a)^
One leg standing time (seconds)		38.83 ± 20.93	18.19 ± 20.72	<0.001(192.5) ^(a)^
ADL independence (Barthel index score)		19.97 ± 0.17	19.76 ± 0.44	0.013(345) ^(a)^
IADL independence (Lawton and Brody score)		23 ± 0.00	21.96 ± 2.67	0.002(332.5) ^(a)^

**Table 4:** Comprehensive model adapted from Lord and Colleagues [1], including measures of frontal and transverse lower limb joint angle complementary model and variables from systematic review by Boyer and Colleagues [5]. Relevant item loadings (>0.7) in bold. Differences between older adults without and with functional disability were assessed with the non-parametric Mann–Whitney U test, with a significance level set at 0.05 and statistical significant differences represented by *.

		Principal Component									
	Gait Parameters	1	2	3	4	5	6	7	8	9	10
Pace	Step time	**0.882**	0.284	−0.224	−0.052	−0.172	0.039	0.112	0.048	−0.095	−0.022
	Stance time	**0.862**	0.29	−0.28	−0.049	−0.182	0.024	0.074	0.057	−0.076	0.056
	Swing time	**0.81**	0.308	−0.086	−0.067	−0.13	0.111	0.178	0.031	−0.117	−0.208
	Step velocity	−0.698	−0.291	0.487	−0.083	0.147	0.125	−0.208	−0.06	−0.033	−0.122
Variability	Step time variability	0.362	**0.865**	−0.171	0.066	−0.169	0.008	0.078	0.067	−0.014	0.103
	Stance time variability	0.357	**0.853**	−0.169	0.076	−0.229	0.01	0.091	0.072	−0.015	0.063
	Swing time variability	0.39	**0.793**	−0.184	0.007	−0.069	0.037	−0.019	0.006	−0.012	0.127
	Step velocity variability	−0.02	**0.771**	−0.281	−0.026	0.03	−0.032	0.209	0.153	0.142	−0.116
Propulsion	Sagittal peak ankle power	−0.179	−0.247	**0.829**	0.044	0.162	0.011	−0.053	0.05	0.003	0.075
	Vertical GRF second peak	−0.303	−0.148	**0.749**	0.025	−0.023	−0.122	−0.074	−0.019	−0.123	−0.21
	AP GRF peak	−0.402	−0.29	**0.714**	−0.031	0.214	0.007	−0.066	−0.015	0.065	0.04
	Sagittal ankle ROM	0.2	−0.044	0.59	0.202	0.343	0.07	−0.374	0.121	0.07	0.354
Hip and knee control	Transverse hip angle at TO	−0.056	0.086	0.068	**0.903**	0.154	−0.008	−0.103	0.108	0.064	0.018
	Transverse hip angle at HS	−0.104	−0.017	0.164	**0.845**	−0.015	−0.117	−0.085	0.031	0.171	−0.019
	Frontal knee angle at TO	0.018	0.027	−0.23	**0.801**	−0.058	0.048	−0.055	−0.002	−0.265	0.078
	Sagittal knee ROM	−0.161	−0.283	0.402	−0.481	0.365	−0.016	−0.117	−0.021	−0.151	0.055
Transverse ankle control	Transverse ankle angle at TO	−0.173	−0.136	0.187	0.011	**0.871**	0.073	−0.048	−0.158	−0.074	0.015
	Transverse ankle angle at HS	−0.136	−0.124	0.059	−0.136	**0.867**	−0.025	0.002	−0.262	−0.209	−0.005
	Transverse knee angle at TO	0.231	0.112	−0.131	−0.477	−0.676	−0.004	0.071	0.025	−0.149	−0.136
Asymmetry	Swing time asymmetry	0.092	−0.135	−0.016	0	0.126	**0.878**	0.262	0.028	0.012	0.029
	Step time asymmetry	−0.151	0	−0.063	−0.138	0.057	**0.873**	−0.04	−0.058	−0.18	0.053
	Stance time asymmetry	0.069	0.39	0.064	0.127	−0.255	**0.704**	0.183	−0.029	0.039	−0.02
	Transverse hip ROM	0.419	−0.064	0.004	−0.064	0.033	0.488	−0.137	0.038	−0.021	−0.07
Sagittal ankle control	Ankle peak plantar flexion	0.125	0.1	−0.181	−0.171	−0.082	0.11	**0.918**	0.07	−0.063	0.041
	Sagittal ankle angle at TO	0.184	0.167	−0.072	−0.071	−0.013	0.134	**0.895**	0.112	−0.041	0.185
Frontal ankle control	Frontal ankle angle at HS	0.011	0.058	0.112	−0.013	−0.142	0.003	0.006	**0.951**	−0.102	0.034
	Frontal ankle angle at TO	0.122	0.147	−0.1	0.134	−0.224	−0.034	0.171	**0.895**	0.093	−0.036
Frontal hip control	Frontal hip angle at TO	−0.028	0.117	−0.064	0.118	0.014	−0.037	−0.043	−0.054	**0.892**	−0.157
	Frontal hip angle at HS	−0.193	−0.071	0.007	−0.117	−0.252	−0.131	−0.07	0.039	**0.792**	0.241
Pre-swing control	Ankle peak dorsiflexion moment	−0.066	−0.162	0.035	−0.164	−0.12	0.04	−0.306	−0.001	0.031	−**0.733**
	Frontal hip ROM	−0.306	−0.196	0.466	−0.152	−0.15	0.055	−0.119	−0.108	−0.034	0.569
	Hip peak extension	−0.228	0.168	−0.284	−0.455	0.079	0.146	0.051	0.153	0.236	0.47
*p*-value of PC scores comparison		0.005 *	0.725	0.297	0.816	0.702	0.770	0.946	0.534	0.004 *	0.946

## Data Availability

Data is unavailable due to privacy.

## Supplementary Materials

The following supporting information can be downloaded at: https://www.mdpi.com/article/10.3390/jfmk10020140/s1, Table S1: Replication of Lord and Colleagues Factor Analysis [1]. Relevant item loadings (>0.7) in bold; Table S2: Complementary factor analysis including lower limb frontal and transverse ROM and positions at HS and TO. Relevant item loadings (>0.7) in bold.

## Abbreviations

The following abbreviations are used in this manuscript:

ADL	Activities of Daily Living
GRF	Ground reaction force
HS	Heel strike
IADL	Instrumental Activities of Daily Living
IPAQ	International Physical Activity Questionnaire
KMO	Kaiser–Meyer–Olkin
MMSE	Mini-Mental State Examination
N	Newton
OLST	One-Leg Standing Test
PC	Principal components
PCA	Principal Component Analysis
PCM	Principal Component Model
ROM	Range of motion
SD	Standard deviation
SRH	Self-reported health
TO	Toe-off
