# Supplementary material for: Kinematic and Kinetic Gait Principal Component Domains in Older Adults With and Without Functional Disability: A Cross-Sectional Study
Source: J Funct Morphol Kinesiol. 2025 Apr 23;10(2):140. doi: 10.3390/jfmk10020140 (PMC12101232; doi:10.3390/jfmk10020140)
Supplement: Supplementary file 1 [file jfmk-10-00140-s001.zip › jfmk-3580736-supplementary.pdf]

Kinematic and Kinetic Gait Principal Component Domains in Older Adults With and Without Functional Disability: A Cross-Sectional Study

Supplementary material

Supplementary Table S1: Replication of Lord and Colleagues Factor Analysis [1]. Relevant item loadings (>0.7) in bold.

KMO=0.647 and Bartlett's Test of Sphericity p<0.001

Gait Spatiotemporal Variable	Pace/Rhythm	Variability	Asymmetry	Postural Control
Step time (s)	**0,943**	0.277	0.068	-0.051
Stance time (s)	**0.928**	0.304	0.031	-0.069
Swing time (s)	**0.862**	0.245	0.179	-0.034
Step velocity (m/s)	**-0.786**	-0.357	0.167	0.421
Step time variability (m/s)	0.415	**0.891**	0.024	0.001
Stance time variability (s)	0.417	**0.880**	0.027	-0.069
Step velocity variability (m/s)	0.012	**0.827**	-0.023	-0.216
Swing time variability (s)	0.445	**0.772**	0.059	0.001
Swing time asymmetry (s)	0.123	-0.152	**0.891**	-0.115
Step time asymmetry (s)	-0.083	-0.056	**0.848**	0.031
Stance time asymmetry (s)	0.098	0.384	**0.735**	-0.091
Step length asymmetry (m)	-0.186	-0.111	-0.255	0.643
Step length (m)	-0.286	-0.457	0.332	0.605
Step length variability (m)	-0.556	0.021	0.317	0.604
Stride width (m)	0.078	-0.007	-0.082	0.604
Variance explained (%)	43.65	16.13	10.88	8.64

Supplementary Table S2: Complementary factor analysis including lower limb frontal and transverse ROM and positions at HS and TO. Relevant item loadings (>0.7) in bold.

KMO=0.535 and Bartlett's Test of Sphericity p<0,001

	PC1	PC2	PC3	PC4	PC5	PC6
Transverse Ankle Angle at TO	**0.909**	-0.046	0.009	-0.21	-0.114	0.019
Transverse Ankle Angle at HS	**0.806**	-0.196	-0.131	-0.329	-0.226	-0.051
Transverse Knee Angle at TO	**-0.801**	-0.437	-0.009	0.053	-0.17	0.139
Transverse Knee Angle at HS	-0.601	-0.471	-0.14	0.081	-0.008	0.433
Transverse Ankle ROM	0.594	0.267	0.367	0.313	-0.082	0.062
Transverse Hip Angle at HS	0.099	**0.880**	0.135	0.009	0.177	-0.192
Transverse Hip Angle at TO	0.221	**0.872**	-0.007	0.122	0.127	0.115
Frontal Knee Angle at TO	-0.106	**0.836**	-0.179	-0.018	-0.286	0.061
Frontal Knee Angle at HS	-0.07	0.588	-0.429	-0.148	-0.542	-0.119
Frontal Hip ROM	0.194	-0.127	**0.866**	-0.029	0.004	-0.036
Frontal Knee ROM	-0.029	0.152	0.646	-0.206	0.289	0.261
Transverse Knee ROM	-0.457	0.055	0.544	0.063	-0.055	-0.331
Frontal Ankle ROM	-0.106	-0.21	0.451	0.49	-0.124	-0.084
Frontal Ankle Angle at HS	-0.074	-0.01	-0.01	**0.930**	-0.049	0.025
Frontal Ankle Angle at TO	-0.223	0.14	-0.157	**0.889**	0.124	0.003
Frontal Hip Angle at TO	-0.064	0.115	-0.18	-0.069	**0.882**	0.013
Frontal Hip Angle at HS	-0.143	-0.099	0.348	0.069	**0.753**	-0.226
Transverse Hip ROM	-0.073	-0.022	0.036	0.003	-0.096	**0.867**
Variance explained (%)	22.89	16.28	14.96	10.34	8.14	6.58

Reference

1. Lord, S.; Galna, B.; Verghese, J.; Coleman, S.; Burn, D.; Rochester, L. Independent domains of gait in older adults and associated motor and nonmotor attributes: validation of a factor analysis approach. *J Gerontol A Biol Sci Med Sci* **2013**, *68*, 820-827, doi:10.1093/gerona/gls255.
